# P-1795. Antimicrobial Stewardship Knowledge, Attitudes, and Practices (KAP) Among Health Care Providers in a Community Hospital Network

**DOI:** 10.1093/ofid/ofae631.1958

**Published:** 2025-01-29

**Authors:** Jeannette Bouchard, April Dyer, Melissa D Johnson, Angelina Davis, Deverick J Anderson, Elizabeth Dodds Ashley

**Affiliations:** DASON, Elgin, South Carolina; Duke Center for Antimicrobial Stewardship and Infection Prevention, Durham, North Carolina; Duke University, Durham, North Carolina; Duke Center for Antimicrobial Stewardship and Infection Prevention, Durham, North Carolina; Duke Center for Antimicrobial Stewardship and Infection Prevention, Durham, North Carolina; Duke Center for Antimicrobial Stewardship and Infection Prevention, Durham, North Carolina

## Abstract

**Background:**

Drivers of inappropriate antimicrobial use (AU) and antimicrobial resistance (AMR) can be linked to deficiencies in prescribers’ knowledge, attitude, and practice (KAP) regarding antibiotics. Currently, KAP information in community hospitals throughout the United States (US) is not well known, and survey information is not standardized. Understanding clinicians’ KAP regarding AU and AMR may provide valuable information for improving antimicrobial stewardship (AS) efforts.

**Figure 1:**
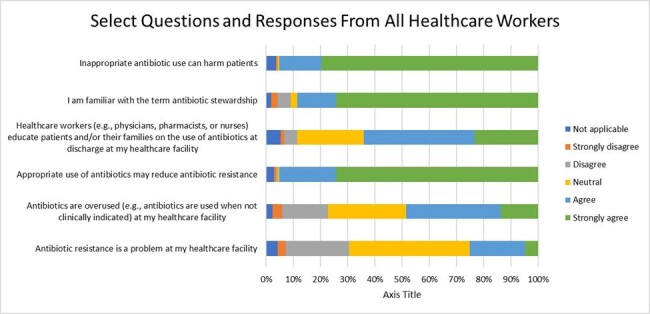

**Methods:**

Prescribers, pharmacists, nurses, and administrators at 40 community hospitals within the Duke Antimicrobial Stewardship Outreach Network (DASON) were invited to take an anonymous, voluntary, 41-question web-based IRB-approved AS KAP survey. This survey is an internationally validated tool translated for use in the US (www.oucru.org/wp-content/uploads/2023/10/KAP_tool.pdf). Responses were collected via REDCap from Feb – Apr 2024.

**Figure 2:**
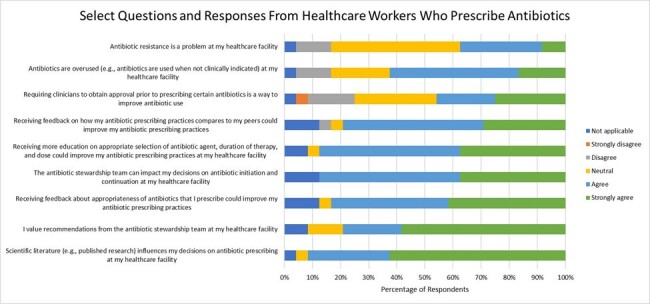

**Results:**

A total of 167 survey responses were received from 24 hospitals; 77 (46%) were pharmacists, 62 (37%) were nurses, and 16 (10%) were physicians. Respondents had a median of 15 years (15.3) of experience in their current healthcare profession and 8.5 years (14.3) in their current institution. Most respondents were familiar with AS (74.3%), strongly agree that antimicrobial overuse can cause harm (79.6%), and that appropriate antibiotic use can reduce AMR (74.3%) (Fig 1). More than half of the respondents who prescribe antibiotics agreed that education and feedback via peer comparison or direct feedback would improve their prescribing (Fig 2). Prescribing respondents also agreed that AS teams impact their antibiotic decisions (87.5%). 63% of prescribers and 46% of non-prescribers agreed antibiotics are overused at their institution. Providers were the most likely to agree AMR is a problem at their institutions (Fig 3).

**Figure 3:**
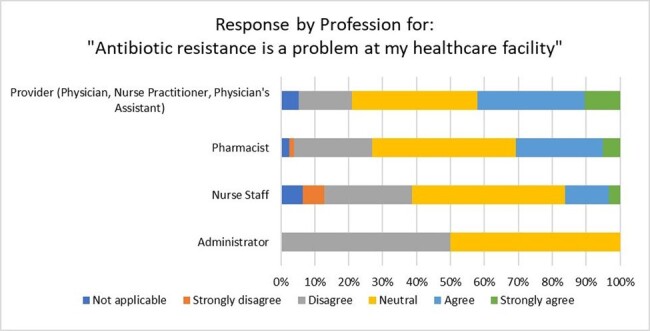

**Conclusion:**

Healthcare workers within DASON were aware of the importance of AS and its effect on AMR and patient outcomes. Prescriber feedback was seen as a valuable educational tool, as most prescribers valued AS feedback and agreed feedback would improve antibiotic selection.

**Disclosures:**

**Melissa D. Johnson, PharmD MHS AAHIVP**, Biomeme: Licensed Technology|Scynexis, Inc: Grant/Research Support|UpToDate: Author Royalties **Elizabeth Dodds Ashley, PharmD, MHS**, HealthtrackRx: Advisor/Consultant|UpToDate: Royalties

